# Digital Literacy and Heart Failure Self-Care in Older Patients and Their Caregivers: Dyadic Analysis Using the Actor-Partner Interdependence Model

**DOI:** 10.2196/85976

**Published:** 2026-03-12

**Authors:** Hokon Kim, Misook L Chung, Yeonsoo Jang, JiYeon Choi, Byung Su Yoo, Sang Hui Chu

**Affiliations:** 1 College of Nursing Yonsei University Seoul Republic of Korea; 2 School of Nursing Vanderbilt University Nashville, TN United States; 3 Mo-Im Nursing Research Institute College of Nursing Yonsei University Seoul Republic of Korea; 4 Yonsei University Institute for Innovation in Digital Healthcare Yonsei University Seoul Republic of Korea; 5 Division of Cardiology Department of Internal Medicine Wonju Severance Christian Hospital Wonju Republic of Korea

**Keywords:** digital literacy, heart failure, self-care, caregiver, symptom perception

## Abstract

**Background:**

The population of patients with heart failure (HF) is rapidly aging, and the prevalence of HF continues to rise among older adults. Effective HF self-care is essential for improving survival and reducing hospital readmissions, and the role of family caregivers in supporting and reinforcing these behaviors has become increasingly important. With the growing integration of digital health technologies into HF management, technology-assisted self-care is becoming more common. However, many older adults experience difficulties in adopting and effectively using digital tools, which may limit the potential benefits of digital health interventions. As both patients’ and caregivers’ levels of digital literacy may jointly shape HF self-care behaviors, a dyadic analytic approach is warranted to clarify their interdependent effects.

**Objective:**

This study aimed to compare digital literacy between older patients with HF and their caregivers and to examine how digital literacy influences HF self-care behaviors within patient-caregiver dyads.

**Methods:**

This cross-sectional study included 102 patients with HF–caregiver dyads recruited from outpatient clinics in South Korea. Digital literacy was measured using the Everyday Digital Literacy Questionnaire. HF self-care, encompassing 3 key dimensions—self-care maintenance, symptom perception, and self-care management—was assessed using the Self-Care of Heart Failure Index (version 7.2) for patients and the Caregiver Contribution to Self-Care of Heart Failure Index (version 2.0) for caregivers. Dyadic associations were analyzed using the Actor-Partner Interdependence Model with maximum likelihood estimation.

**Results:**

Patients (age: mean 79.4, SD 9.1 years) had significantly lower digital literacy than caregivers (age: mean 59.0, SD 13.1 years). Caregivers primarily consisted of adult children (63/102, 61.8%), followed by spouses (33/102, 32.4%). The Actor-Partner Interdependence Model results revealed significant actor effects of digital literacy on symptom perception for both patients (β=0.26; *P*=.008) and caregivers (β=0.32; *P*=.002). For self-care management, a significant actor effect was observed only for patients (β=0.24; *P*=.02). No significant actor effects were found for self-care maintenance, and no partner effects reached statistical significance across any dimension.

**Conclusions:**

Digital literacy significantly influenced individuals’ own HF self-care behaviors, particularly symptom perception, but cross-partner effects were not observed within dyads. These findings suggest that digital health interventions should assess and address patients’ and caregivers’ digital skills as distinct targets rather than assuming spillover effects within dyads. To optimize HF outcomes in aging populations, culturally sensitive and dyad-focused strategies that consider individual digital literacy are essential.

## Introduction

Heart failure (HF) is a prevalent and progressive cardiovascular syndrome among older adults, affecting approximately 18.6% of adults aged 80 years and older in Korea [1]. It is driven by age-related structural and functional changes in the cardiovascular system as well as cumulative cardiac diseases across the life course [2]. Older patients with HF often present with multiple comorbidities, frailty, and functional limitations, making disease management increasingly complex [3]. As populations age globally, the burden of HF continues to rise with high readmission and mortality rates among older adults, resulting in substantial health care use and economic burden [1,2,4,5].

Effective self-care is central to slowing HF progression, preventing irreversible cardiac damage, and improving long-term outcomes [5-7]. Substantial evidence consistently demonstrates that appropriate HF self-care reduces hospital readmissions and mortality and enhances the quality of life among patients with HF [8-10]. HF self-care is theoretically grounded in situation-specific theories of HF management and conceptualized as a multidimensional process encompassing self-care maintenance, symptom perception, and self-care management. These dimensions enable patients to maintain physiological stability, detect changes in symptoms, and respond appropriately to exacerbations [6,7,11]. Particularly, symptom perception—the ability to recognize, interpret, and evaluate bodily changes—has gained increasing attention because of its strong association with lower mortality and fewer readmissions [4,12,13].

Despite its importance, effective self-care is particularly challenging for older adults with HF [3]. Cognitive decline, physical limitations, multimorbidity, and reduced self-efficacy often hinder adherence to recommended behaviors and delay timely symptom perception and management [8,12,14,15]. In the context of aging, successful self-care increasingly depends on external support, as age-related functional decline limits patients’ ability to independently manage complex treatment regimens [3,14,16]. This interdependence highlights the essential role of caregivers in facilitating HF self-care among older adults [3,17]. Caregivers assist with medication management, symptom monitoring, and decision-making, compensating for cognitive or functional limitations [3,16]. Consequently, HF self-care in later life should be conceptualized as a dyadic process embedded within patient-caregiver relationships rather than as isolated individual efforts and behaviors [17-19].

As HF management increasingly incorporates digital health technologies—including mobile apps, telemonitoring systems, and wearable devices—these tools have become integral components of contemporary care, enabling remote monitoring and promoting patient-centered self-care beyond traditional clinical settings [20,21]. However, the effectiveness of digital health technologies depends largely on users’ acceptance and digital literacy [22,23], particularly among older adults who often experience difficulties with technology adoption and use [24,25].

Digital literacy, defined as the ability to access, understand, evaluate, and apply digital information, is a key determinant of successful engagement with digital health interventions [22]. Older adults generally exhibit lower levels of digital literacy than younger populations, which can limit effective technology use, undermine self-care adherence, and exacerbate digital health disparities in HF care [23-25]. Because caregivers frequently assist older patients in navigating digital environments [26,27], understanding digital literacy within patient-caregiver dyads is essential for developing inclusive and sustainable HF self-care strategies [3,17].

Given the frequent and ongoing interactions between patients and caregivers in older populations, analyses focused solely on individuals are insufficient to capture the complexity of HF self-care processes [3,17,18]. A dyadic analytic framework, such as the Actor-Partner Interdependence Model (APIM), allows the simultaneous evaluation of how individual characteristics influence both one’s own outcomes (actor effects) and those of one’s partner (partner effects), capturing bidirectional dynamics that are often overlooked in individual-level analyses [19,28].

Importantly, patient-caregiver relationships vary substantially across cultural contexts [18,29]. While previous research in Western settings has predominantly focused on spousal caregivers [30-32], East Asian societies, influenced by Confucian values, emphasize family-centered caregiving, with adult children frequently assuming the primary caregiving role [18,29]. Despite these cultural distinctions, empirical research examining dyadic processes and the role of digital literacy in HF self-care within Asian contexts remains remarkably scarce [19]. This gap is especially salient in East Asian contexts, where family-centered caregiving and age-related digital literacy challenges frequently coexist.

Therefore, this study aimed to (1) compare digital literacy levels between older patients with HF and their caregivers and (2) examine how digital literacy influences HF self-care behaviors within patient-caregiver dyads using a dyadic analytic framework. By identifying actor and partner effects, this study seeks to inform the design of culturally sensitive, dyad-focused digital health interventions that enhance collaborative HF self-care among older adults with HF and their caregivers in Korea and similar East Asian contexts.

## Methods

### Participants and Study Design

This cross-sectional study recruited 102 dyads of patients with HF and their caregivers from the outpatient clinic of a tertiary hospital in South Korea between March and September 2023. Eligible patients were adults aged 19 years or older with a confirmed diagnosis of HF. Caregivers were adults aged 19 years or older who provided unpaid care to the participating patients. Patients were excluded if they had a history of heart transplantation or left ventricular assist device implantation or had cognitive impairment that could interfere with study participation. Caregivers who were paid or formally employed by the patient or the patient’s family were also excluded.

### Ethical Considerations

This study was approved by the institutional review board of Yonsei University Health System (approval 4-2022-1622). All participants provided written informed consent before participation. The survey was conducted anonymously, and no personally identifiable information was collected. As compensation for participation, both patients and their caregivers received a small gift equivalent to approximately US $7.

### Measurements

#### Digital Literacy

Digital literacy was assessed using the Everyday Digital Literacy Questionnaire (EDLQ), a 22-item instrument developed and validated for older adults in South Korea [33]. The EDLQ measures 3 domains: information and communication (9 items), content creation and management (4 items), and safety and security (9 items). Items are rated on a 5-point Likert scale ranging from 1 (strongly disagree) to 5 (strongly agree), with higher total scores indicating greater overall digital literacy. The EDLQ has demonstrated excellent internal consistency, with a Cronbach α of 0.98 at the time of development [33] and 0.96 in this study.

#### HF Self-Care and Caregiver Contribution to HF Self-Care

Patients’ HF self-care was assessed using the Self-Care of Heart Failure Index (version 7.2), a 29-item instrument comprising 3 dimensions: self-care maintenance (11 items), symptom perception (10 items), and self-management (8 items). Items are rated on a 5-point Likert scale. Following the scoring guidelines provided by the instrument developers, raw scores of each dimension were transformed into standardized scores ranging from 0 to 100, with higher standardized scores indicating better self-care. The Self-Care of Heart Failure Index (version 7.2) was originally developed by Riegel et al [6] and has been culturally adapted and validated for use in Korea [34]. Reported internal consistency ranges from Cronbach α of 0.70 in the original version [6] to 0.71 to 0.96 in the Korean version [34]. In this study, Cronbach α values for patients’ HF self-care ranged from 0.76 to 0.84 across dimensions. Additionally, the caregiver contribution to HF self-care was measured using the Caregiver Contribution to Self-Care of Heart Failure Index (version 2.0), a parallel instrument designed to assess caregivers’ support for patients’ HF self-care across the same 3 dimensions. Scores were standardized on a 0 to 100 scale. The Caregiver Contribution to Self-Care of Heart Failure Index (version 2.0) has demonstrated strong validity in previous studies (Cronbach α=0.90) [35]. In this study, Cronbach α values for caregiver contribution were 0.72 for maintenance, 0.85 for symptom perception, and 0.76 for self-care management.

#### Covariates

Dyadic mutuality was assessed using the 15-item Mutuality Scale from the Family Caregiving Inventory, which evaluates love, shared values, reciprocity, and enjoyable activities on a 5-point scale. Higher scores indicate greater perceived mutuality [36]. The scale has demonstrated strong internal consistency in previous studies (Cronbach α=0.91-0.96) [31,37] and in this study (Cronbach α=0.95). Although mutuality was assessed for both dyad members, patient and caregiver scores were highly correlated (*r*>0.70; *P*<.001). To maintain model parsimony and avoid multicollinearity within the APIMs [38], only caregiver-perceived mutuality was included as a covariate, reflecting the caregiver’s central role in shaping HF self-care support and caregiving behaviors [37,39]. Caregiver relationship type (spouse vs adult children) was included to account for differences in caregiving roles and responsibilities [29,30]. Patient comorbidity burden was assessed using the age-adjusted Charlson Comorbidity Index (ACCI), which incorporates age as a weighted component in addition to comorbid conditions to better capture overall disease complexity in older adults [40]. Additionally, patients’ perceived economic status and residential areas (urban vs rural) were included to capture potential socioeconomic and contextual disparities in digital literacy, health resource access, and HF self-care behaviors in older adults [22,24,25].

### Data Analysis

Data analyses were conducted using SPSS Statistics (version 26.0; IBM Corp) and SPSS AMOS (version 30.0; IBM Corp). Descriptive statistics were used to summarize participant characteristics and study variables.

### Preliminary Analyses and Assumptions

Before model estimation, distributional assumptions for all primary continuous variables were examined using skewness and kurtosis statistics, histogram inspection, and normal *Q-Q* plots. These diagnostic procedures were conducted to assess the suitability of maximum likelihood (ML) estimation for APIM-based structural equation models. Although several variables demonstrated mild departures from normality—common for bounded Likert-type measures in behavioral and health research—all values remained within acceptable ranges for ML estimation in structural equation modeling (|skewness|<2.0; |kurtosis|<7.0) [41-43]. No evidence of severe nonnormality or influential outliers was identified. Given these findings and the moderate sample size (102 dyads), no variable transformations were applied, and ML estimation was retained for all analyses.

Bivariate associations among key variables were examined using Pearson correlation coefficients for continuous variables. Spearman correlations were applied to assess the association involving the ordinal variables (ie, perceived economic status). A complete correlation matrix with exact sample size, correlation coefficients, and *P* values is presented in Multimedia Appendix 1.

### APIM Model Specification

Dyadic relationships were analyzed using the APIM, treating dyad members as a priori distinguishable by role (patients with HF vs caregivers) [38]. Each dyad consisted of 1 patient and 1 identified primary caregiver, recruited and coded separately by role. Digital literacy was measured using the same instrument for both members, whereas HF self-care outcomes were assessed using parallel role-specific instruments (patient HF self-care vs caregiver contribution to HF self-care).

Separate APIMs were estimated for each HF self-care dimension (eg, maintenance, symptom perception, and management) using ML estimation in AMOS. In each model, four structural paths were freely estimated: (1) two actor effects (patient digital literacy predicting patient HF self-care and caregiver digital literacy predicting caregiver contribution to HF self-care) and (2) two partner effects (patient digital literacy predicting caregiver contribution to HF self-care and caregiver digital literacy predicting patient HF self-care).

Patient and caregiver digital literacy scores, specified as the primary predictors, were allowed to covary to account for interdependence within dyads. Residuals of patient HF self-care and caregiver contribution to HF self-care were also correlated to capture dyadic interdependence not explained by the predictors. Five covariates—caregiver-perceived mutuality, caregiver relationship type (spouse vs adult child), patient comorbidity burden (ACCI), patient’s perceived economic status, and residential area—were specified as exogenous variables, and covariances among all exogenous variables were freely estimated. No equality constraints were imposed across patient and caregiver paths. APIMs were estimated using a structural equation modeling framework to account for dyadic interdependence. Consistent with previous APIM studies [44], global model fit indices were not reported because the model was saturated. Model evaluation focused on the magnitude, direction, and statistical significance of estimated actor and partner effects, covariance, and explained variance (*R*^2^) for patient and caregiver outcomes. The analyses represent concurrent (cross-sectional) APIMs. No mediators were specified, and no indirect effects were estimated.

## Results

### Characteristics of Patients With HF and Their Caregivers

A total of 102 patient-caregiver dyads were included in the analysis. Patients with HF had a mean age of 79.4 (SD 9.1) years, and 55 (53.9%) patients were female. Caregivers were younger (age: mean 59.0, SD 13.1 years) and predominantly female (n=67, 65.7%). Most caregivers were adult children, including sons, daughters, daughters-in-law, and sons-in-law (n=63, 61.7%), followed by spouses (n=33, 32.4%). Nearly half (n=49, 48%) of the patients lived with their caregivers. Patients generally had lower educational attainment compared with caregivers, and half (n=51, 50%) of the patients resided in rural areas.

Mean mutuality score indicated moderate levels of perceived mutuality for both patients (mean 2.8, SD 0.7) and caregivers (mean 2.7, SD 0.9). Patient and caregiver mutuality were strongly correlated (*r*=0.70; *P*<.001; Multimedia Appendix 1), reflecting the interdependent nature of patient-caregiver dyads.

Regarding clinical characteristics, 55 (53.9%) patients had HF with preserved ejection fraction. The mean left ventricular ejection fraction was 52.25% (SD 12.2%). Most patients presented with mild HF symptoms, classified as New York Heart Association class I to II, accounting for 89 (87.3%) samples. Patients exhibited a substantial comorbidity burden, with a mean ACCI score of 6.4 (SD 2.2). Detailed demographic and clinical characteristics are summarized in Table 1.

**Table 1:** Characteristics of patients with heart failure and their caregivers^a^.

Characteristics		Patients (n=102)	Caregivers (n=102)
Age (years), mean (SD)		79.4 (9.1)	59.0 (13.1)
Female, n (%)		55 (53.9)	67 (65.7)
**Education level, n (%)**			
	Below elementary school	67 (65.7)	19 (18.6)
	Middle-to-high school	30 (29.4)	48 (47.1)
	Above college	5 (4.9)	35 (34.3)
**Perceived economic status, n (%)**			
	Low	65 (63.7)	30 (29.4)
	Moderate to high	37 (36.3)	72 (70.6)
Residential area (urban), n (%)		51 (50.0)	—^b^
**Relationship type,** **n** **(%)**			
	Spouse	—	33 (32.4)
	Adult child	—	63 (61.7)
Living arrangement (cohabitation), n (%)		49 (48.0)	—
**New York Heart Association class,** **n** **(%)**			
	Class I-II (mild symptoms)	89 (87.3)	—
	Class III (moderate symptoms)	13 (12.7)	—
**Left ventricular ejection fraction (%), mean (SD)**		52.3 (12.2)	—
	≤40% (heart failure with reduced ejection fraction), n (%)	18 (17.6)	—
	41%-49% (heart failure with mildly reduced ejection fraction), n (%)	29 (28.4)	—
	≥50% (heart failure with preserved ejection fraction), n (%)	55 (53.9)	—
Comorbidity (age-adjusted Charlson Comorbidity Index), mean (SD)		6.4 (2.2)	—
Mutuality score, mean (SD)		2.8 (0.7)	2.7 (0.9)

^a^Patients and caregivers are members of the same dyad; therefore, values are presented for descriptive purposes only.

^b^Not applicable.

### Digital Literacy and HF Self-Care Outcomes Within Dyads

Patients demonstrated significantly lower digital literacy than caregivers (mean 31.9, SD 20.6 vs mean 77.8, SD 30.8: *P*<.001). HF self-care outcomes were assessed across 3 dimensions: maintenance, symptom perception, and management. Symptom perception was the lowest-scoring dimension for both patients’ HF self-care (mean 46.9, SD 16.6) and caregivers’ contribution to HF self-care (mean 52.5, SD 19.7). Descriptive statistics of HF self-care outcomes across the 3 dimensions are presented in Table 2.

**Table 2:** Comparisons of digital literacy and heart failure (HF) self-care outcomes between patients and caregivers (N=102 dyads)^a^.

Variables		Patients, mean (SD)	Caregivers, mean (SD)	*t* test (*df*)	*P* value
**Digital literacy**					
	Total score	31.9 (20.6)	77.8 (30.8)	–12.60 (101)	<.001
	Safety and security	13.5 (9.5)	33.3 (12.9)	–12.43 (101)	<.001
	Information and communication	13.0 (8.1)	31.9 (12.6)	–12.98 (101)	<.001
	Contents creation and management	5.4 (3.3)	12.6 (6.1)	–10.51 (101)	<.001
**HF self-care**					
	Maintenance	62.6 (10.2)	59.0 (17.4)	2.05 (101)	.04
	Symptom perception	46.6 (16.6)	52.5 (19.7)	–2.66 (101)	.009
	Management	62.1 (16.1)	65.0 (18.1)	–1.39 (101)	.17

^a^Comparisons between patients and caregivers were conducted using paired *t* tests (2-tailed), as they were members of the same dyad.

### Dyadic Associations Between Digital Literacy and HF Self-Care (APIM Results)

**Figure 1:**
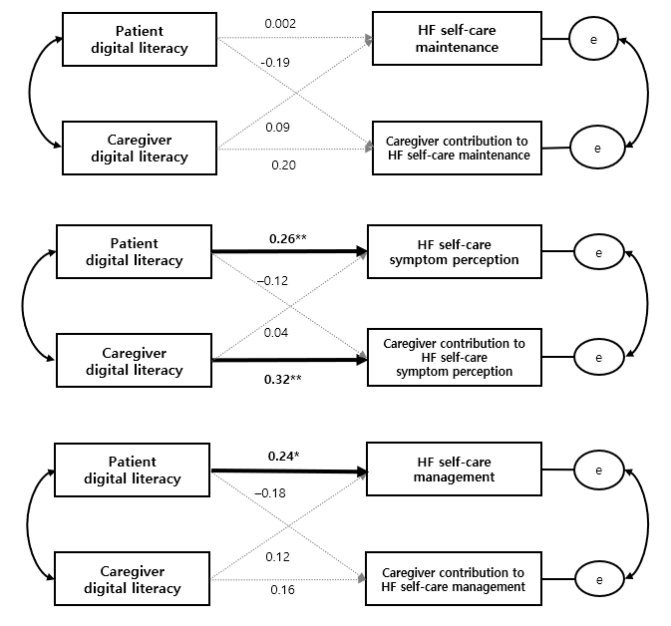
Actor-Partner Interdependence Model examining the associations between patient and caregiver digital literacy and heart failure (HF) self-care outcomes across 3 dimensions: HF self-care maintenance, HF self-care symptom perception, and HF self-care management. Solid arrows indicate statistically significant actor effects, whereas dashed arrows indicate nonsignificant paths. Standardized coefficients (β) are shown. *P<.05, **P<.01.

Across the 3 HF self-care dimensions, distinct patterns of association between digital literacy and self-care outcomes were observed. For HF self-care maintenance, digital literacy was not significantly associated with patient or caregiver outcomes, indicating no interpretable actor-partner structure in this dimension. In contrast, for symptom perception, significant actor effects of digital literacy were observed for both members of the dyad. Higher patient digital literacy was associated with better patient symptom perception (B=0.21; β=0.26, SE 0.08, 95% CI 0.06-0.37; *P*=.008), and higher caregiver digital literacy was associated with greater caregiver contribution to symptom perception (B=0.21; β=0.32, SE 0.07, 95% CI 0.08-0.34; *P*=.002), reflecting an actor-dominant pattern with no evidence of cross-partner effects. For self-care management, only the patient’s digital literacy demonstrated a significant actor effect on patient self-care outcome (B=0.19; β=0.24, SE 0.08, 95% CI 0.03-0.35; *P*=.02), whereas no other actor or partner effects reached statistical significance for either member of the dyad. Detailed APIM results are presented in Table 3. The model accounted for approximately 22.6% of the variance in patients’ symptom perception, 35.3% in caregivers’ symptom perception, and 14.6% in patients’ self-care management. A summary of standardized actor and partner effects across the 3 APIMs is presented in Figure 1.

**Table 3:** Actor and partner effects of digital literacy on heart failure (HF) self-care based on the Actor-Partner Interdependence Model (N=102 dyads).

Outcome or effect, role				B		β (SE; 95% CI)		*P* value
**HF self-care maintenance**								
	**Actor effect**							
		Patient	0.001		0.002 (0.05; –0.10 to 0.11)		.98	
		Caregiver	0.11		0.20 (0.06; –0.01 to 0.24)		.08	
	**Partner effect**							
		Patient→caregiver	–0.16		–0.19 (0.08; –0.32 to 0.004)		.06	
		Caregiver→patient	0.03		0.09 (0.04; –0.05 to 0.11)		.48	
**HF self-care symptom perception**								
	**Actor effect**							
		Patient	*0.21* ^a^		*0.26 (0.08; 0.06 to 0.37)*		*.008*	
		Caregiver	*0.21*		*0.32 (0.07; 0.08 to 0.34)*		*.002*	
	**Partner effect**							
		Patient→caregiver	–0.11		–0.12 (0.09; –0.29 to 0.06)		.19	
		Caregiver→patient	0.02		0.04 (0.06; –0.10 to 0.14)		.73	
**HF self-care management**								
	**Actor effect**							
		Patient	*0.19*		*0.24 (0.08; 0.03 to 0.35)*		*.02*	
		Caregiver	0.09		0.16 (0.07; –0.03 to 0.22)		.15	
	**Partner effect**							
		Patient→caregiver	–0.15		–0.18 (0.08; –0.32 to 0.01)		.07	
		Caregiver→patient	0.06		0.12 (0.06; –0.06 to 0.18)		.34	

^a^Italicized values indicate statistically significant effects (*P*<.05).

## Discussion

### Principal Findings

To the best of our knowledge, this is the first study to examine the influence of digital literacy on HF self-care using APIM within patient-caregiver dyads. Significant actor effects of digital literacy were found for both patients and caregivers on the symptom perception dimension of HF self-care, whereas no partner effects were observed. Patients with higher digital literacy demonstrated a better ability to detect and interpret symptoms and to engage in effective self-care management. Likewise, caregivers with higher digital literacy showed greater competence in identifying HF-related changes, although their digital literacy did not significantly affect patients’ overall maintenance or management behaviors. These findings highlight the independent role of digital literacy in shaping HF self-care behaviors and underscore the need for strategies that enhance digital literacy in both patients and caregivers [45].

Previous research indicates that educational attainment and access to health information are closely related to both digital literacy and HF self-care, particularly symptom perception [12,15,46]. Lower educational levels have been linked to reduced self-care autonomy and delayed recognition of symptom changes, often through the pathway of limited digital literacy [22,25]. In this study, the generally low education level of the older patients, combined with the fact that half of the participants (51/102, 50%) resided in rural areas, likely reflects a broader digital divide [22,25]. These sociocontextual factors may help explain the overall low digital literacy observed in our sample and emphasize the persistent digital disparities faced by older adults with HF [22,23,25].

In this context, caregivers with higher digital literacy demonstrated greater contributions to symptom perception, enabling faster identification of changes in patients’ health status [3,11,12,47]. However, contrary to our initial hypothesis, caregivers’ digital literacy did not directly affect maintenance or management dimensions of HF self-care. These findings may also reflect the current design orientation of many digital health tools for HF, which predominantly emphasize information provision and symptom monitoring rather than sustained lifestyle modification [20,21,48,49]. Accordingly, digital literacy may facilitate symptom perception without necessarily translating into long-term behavioral change in HF self-care maintenance and management [50].

This finding may also be explained by entrenched lifestyle patterns among older adults, such as long-standing dietary and physical activity habits that are less amenable to behavioral change [8,16]. Furthermore, as most caregivers in this study were patients’ adult children, their capacity to significantly alter patients’ long-standing lifestyle patterns—especially regarding diet and physical activity routines—is likely limited within the context of family-centered caregiving norms in East Asian societies [18,29].

These cultural caregiving norms are particularly evident in Korea. In many East Asian societies shaped by Confucian values that prioritize filial piety, adult children frequently take primary responsibility for caregiving [18,29]. However, rapid industrialization and changing social structures have reduced opportunities for intergenerational coresidence, resulting in caregiving that is frequently provided without daily cohabitation [18,51]. In such contexts, caregivers may play more of a technical or assisting role—helping with device use or symptom monitoring—rather than engaging in continuous motivational or educational support [26,27]. This relational pattern may reduce the potential for partner effects on self-care behaviors. This finding contrasts with previous research that has emphasized caregiver support—particularly within spousal dyads—as an important determinant of adherence [9,31,32].

Finally, we adjusted for mutuality—the quality of reciprocal understanding and rapport between patient and caregiver—to isolate the effects of digital literacy [31,36]. Because mutuality is known to influence communication, decision-making, and joint self-care [31,36,37,39], future studies should explore how digital literacy and mutuality interact over time or across caregiving stages to shape dyadic engagement in digital health–based management.

### Limitations

Despite the valuable insights provided by this study, several limitations should be noted. First, caregiving in South Korea often involves multiple family members rather than a single designated caregiver, which may limit the transferability of the findings. Second, the study was conducted in a single center with mixed urban and rural characteristics, and the limited sample size may affect generalizability. Third, other relevant factors, including cognitive function and depressive symptoms, were not examined and should be considered in future dyadic studies of digital health–supported self-care. Finally, the reliance on self-reported data may have introduced bias; future studies should incorporate objective measures of digital literacy, self-care behaviors, and clinical outcomes (eg, hospitalization or mortality) to better assess health impact.

### Conclusions

This dyadic analysis indicates that digital literacy plays a significant role in HF self-care, particularly in enhancing symptom perception for both patients and caregivers, while cross-partner influences were not evident. The findings suggest that digital literacy interventions should engage both members of the dyad, with strategies tailored to caregiving relationships, cultural norms, and individual readiness. To bridge the gap between symptom perception and behavior change, future digital health interventions should integrate more interactive, habit-forming features beyond simple monitoring. In addition, considering older adults’ ingrained habits, mutuality, and sociocultural caregiving patterns will be essential in designing effective, equitable digital self-care programs. Future longitudinal and intervention studies are needed to validate these pathways and optimize collaborative self-care in diverse populations with HF.

## Data Availability

The datasets generated or analyzed during this study are available from the corresponding author on reasonable request.

## Appendix

Multimedia Appendix 1 Bivariate correlations among key variables (N=102 dyads).

## Abbreviations

ACCI: age-adjusted Charlson Comorbidity Index
APIM: Actor-Partner Interdependence Model
EDLQ: Everyday Digital Literacy Questionnaire
HF: heart failure
ML: maximum likelihood
