# Supplementary material for: Digital Literacy and Heart Failure Self-Care in Older Patients and Their Caregivers: Dyadic Analysis Using the Actor-Partner Interdependence Model
Source: JMIR Aging. 2026 Mar 12;9:e85976. doi: 10.2196/85976 (PMC13022548; doi:10.2196/85976)
Supplement: Multimedia Appendix 1 [file aging_v9i1e85976_app1.docx]

**Bivariate correlations among key variables (*n*=102 dyads).**

| **Variable** | **1** | **2** | **3** | **4** | **5** | **6** | **7** | **8** | **9** | **10** | **11** | **12** |
| --- | --- | --- | --- | --- | --- | --- | --- | --- | --- | --- | --- | --- |
| 1. Patient digital literacy | 1 |  |  |  |  |  |  |  |  |  |  |  |
| 2. Caregiver digital literacy | .02 | 1 |  |  |  |  |  |  |  |  |  |  |
| 3. Patient self-care maintenance | .04 | .09 | 1 |  |  |  |  |  |  |  |  |  |
| 4. Patient symptom perception | .33** | .09 | .31** | 1 |  |  |  |  |  |  |  |  |
| 5. Patient self-care management | .28** | .13 | .58** | .48** | 1 |  |  |  |  |  |  |  |
| 6. Caregiver contribution: maintenance | .02 | .03 | .28** | .05 | .26** | 1 |  |  |  |  |  |  |
| 7. Caregiver contribution: symptom perception | .09 | .24* | .23* | .34** | .33** | .56** | 1 |  |  |  |  |  |
| 8. Caregiver contribution: management | .01 | .12 | .33** | .18 | .27** | .67** | .50** | 1 |  |  |  |  |
| 9. Patient mutuality | .04 | .17 | .22* | .37** | .26** | .12 | .37** | .29** | 1 |  |  |  |
| 10. Caregiver mutuality | .14 | .16 | .28** | .35** | .26** | .32** | .49** | .43** | .70** | 1 |  |  |
| 11. Patient perceived economic status† | .36** | .07 | −.01 | .19 | .11 | .22* | .26** | .26** | .13 | .15 | 1 |  |
| 12. Caregiver perceived economic status† | .22* | .41** | −.08 | .09 | .08 | −.04 | .14 | .01 | −.03 | .06 | .39** | 1 |

Note. Pearson correlation coefficients were used for associations between continuous variables.

Spearman rank-order correlations were used for associations involving ordinal variables († perceived economic status for patients and caregivers).

All correlations were based on n=102 dyads.

*P<.05; **P<.01 (two-tailed).
